# Estimating Diarrhea Mortality among Young Children in Low and Middle Income Countries

**DOI:** 10.1371/journal.pone.0029151

**Published:** 2012-01-03

**Authors:** Christa L. Fischer Walker, Martin J. Aryee, Cynthia Boschi-Pinto, Robert E. Black

**Affiliations:** 1 Department of International Health, Johns Hopkins Bloomberg School of Public Health, Baltimore, Maryland, United States of America; 2 Division of Biostatistics and Bioinformatics, Department of Oncology, Johns Hopkins University School of Medicine, Baltimore, Maryland, United States of America; 3 Department of Child and Adolescent Health and Development, World Health Organization, Geneva, Switzerland; 4 Universidade Federal Fluminense, Rio de Janeiro, Brazil; University of Cape Town, South Africa

## Abstract

**Background:**

Diarrhea remains one of the leading causes of morbidity and mortality among children under 5 years of age, but in many low and middle-income countries where vital registration data are lacking, updated estimates with regard to the proportion of deaths attributable to diarrhea are needed.

**Methods:**

We conducted a systematic literature review to identify studies reporting diarrhea proportionate mortality for children 1–59 mo of age published between 1980 and 2009. Using the published proportionate mortality estimates and country level covariates we constructed a logistic regression model to estimate country and regional level proportionate mortality and estimated uncertainty bounds using Monte-Carlo simulations.

**Findings:**

We identified more than 90 verbal autopsy studies from around the world to contribute data to a single-cause model. We estimated diarrhea proportionate mortality for 84 countries in 6 regions and found diarrhea to account for between 10.0% of deaths in the Americas to 31.3% of deaths in the South-east Asian region.

**Discussion:**

Diarrhea remains a leading cause of death for children 1–59 mo of age. Published literature can be used to create a single-cause mortality disease model to estimate mortality for countries lacking vital registration data.

## Introduction

Although child survival rates have made improvements in the past decade, 8.8 million children still die every year before reaching their 5^th^ birthday [1]. As mortality rates continue to decline it is important to better understand the causes of the remaining child deaths. Among low and middle income countries, where 94% of child deaths occur, vital registration data are largely incomplete or missing entirely [2]. Hence, estimates based on post-mortem interviews with family members, generally referred to as verbal autopsy methods, and are critical to fill in large knowledge gaps.

The Child Health Epidemiology Reference Group (CHERG) has developed standards for systematic literature reviews and a cross validation process including both single and multi-cause modeling approaches for estimating causes of child deaths where vital registration data are not available [3], [4]. The CHERG recently published 2008 estimates for all causes of child death, including diarrhea [5] utilizing a multi-cause modeling approach for children 1–59 mo of age. As part of the CHERG review process, Boschi-Pinto et al estimated diarrhea-specific proportionate mortality accounted for 19% of child deaths in 2000 and thus when applied to the total deaths in 2004, diarrhea was responsible for approximately 1.8 million deaths among children under 5 years of age in low and middle income countries [4]. Since this publication, the overall number of child deaths has declined by more than 1.5 million and new cause-specific mortality data have become available, thus it is necessary to update the review of the literature, refine the CHERG analytical methodology and produce more current diarrhea mortality estimates for world regions.

We conducted a systematic review of all papers reporting diarrhea proportionate mortality among children less than 5 years of age with the objective of estimating diarrhea proportionate mortality. We thoroughly reviewed the child diarrhea mortality literature and utilize 2008 country-specific indicators to produce diarrhea mortality estimates for countries where vital registration data are not available based on a single cause modeling approach. This methodological alternative has the benefit of using all possible diarrhea-specific data and thus provides an important comparison to the previously published multi-cause approach.

## Methods

We conducted a systematic review of the published literature to identify studies with diarrhea as a cause of death among children less than 5 years of age. We searched PubMed, CAB abstracts, the WHO Regional Databases, and SIGLE for all studies containing data on diarrhea proportionate mortality published between 1980 and 2008 using all combinations of “diarrh(o)ea”, “mortality”, and “cause of death” both as keywords and MeSH terms. Studies conducted in low and middle-income countries and published in all languages were included. We included community based mortality studies with at least 12 months of mortality monitoring. All included studies used standardized verbal autopsy methodologies. Studies were judged to be of poor quality and unsuitable for inclusion if methods were poorly described, if the study was conducted in a non-representative population (such as low birth weigh infants), or if the cause of death was attributable to more than one cause unless the death could be proportionately reallocated to single causes. Because we sought to estimate diarrhea proportionate mortality for all children 1–59 mo of age we excluded verbal autopsy studies conducted among non-standardized age groups (i.e. those with upper age limits other than 11/12 mo, 35/36 mo, or 59/60 mo). Data from included studies were abstracted into a standard data abstraction sheet by two trained abstractors and all discrepancies were resolved by one of the study investigators. To minimize bias we also sought unpublished data from investigators who were able to provide a full report of study methods and results.

Our outcome of interest was diarrhea proportionate mortality. When studies did not report proportionate mortality, this was calculated using diarrhea-specific and all-cause mortality rates. For studies that attributed a proportion of deaths to multiple causes, these deaths were distributed to single causes according to the relative single cause proportions. Because we did not find representative mortality studies for all countries of the world where vital registration data are lacking, we used study and country level covariates to create a regression model to predict diarrhea proportionate mortality for all countries lacking vital registration data. We chose a logistic model because it allows modeling of the outcome variable with estimates constrained between 0 and 1. Observations were weighted by the square root of sample size to prevent studies with extremely large samples sizes from overweighting the model. The model included under 5 mortality risk (5q0), malaria risk, Gross National Income (GNI), year of data collection, and WHO region (Table 1). We abstracted the author- reported site-specific under 5 mortality risk (5q0) per 1,000 live births when possible. For studies where 5q0 was not reported, but under 5 or under 1 mortality rates were reported, these were transformed using standard life tables [6]. For studies that did not report 5q0 or any overall all-cause mortality indicators, we used under 5 mortality risks reported in the Demographic and Health Surveillance Surveys or the WHO Core Indicators Tables. Because the cause of death profile in Africa can be influenced by malaria endemicity, for countries in Africa, we estimated country level malaria risk with a covariate that combined MARA endemicity maps [7] and Guerra's population at risk [8]. As a proxy for the national wealth index we used GNI expressed as purchasing power parity. Malaria risk and 5q0 were insignificant predictors and fell out of the model. Risk of mortality (5q0) is often a strong predictor in cause of death models, but here 5q0 and GNI were strongly correlated and GNI resulted in a better model fit, thus it was retained and 5q0 released from the model. We used WHO regions to ensure our results are comparable to previously published diarrhea mortality estimates [4], [5].

**Table 1:** Logistic regression model parameter estimates.

Parameter	Estimate
WHORegion AFR	−9.29E−01
WHORegion AMR	−1.95E−01
WHORegion EMRSEAR	−5.96E−01
WHORegion WPR	−1.60E+00
GNI	−1.70E−04
Post 1990	−1.16E−01
lowAge 0	−2.69E−01
lowAge 2	−2.66E−01
highAge 1	−2.22E−01
highAge 2	3.00E−01

Our analysis sought to estimate the proportion of mortality attributable to diarrhea among children 1–59 mo of age; however, some studies included neonatal deaths that could not be excluded during the data abstraction period. Because diarrhea deaths are rare in the neonatal period, we included a neonatal dummy variable in the model to help account for the residual effect of these neonatal deaths being included in the final model. Similarly, we sought to estimate diarrhea mortality for children up to 59 mo of age. However, many studies included only infants up to 12 or 36 mo of age. Thus, we created dummy variables to differentiate studies that included children up to the full 59 mo of age from those with truncated age cohorts up to 12 or 36 mo of age.

Time was included as a dichotomous interaction variable with the AMR region differentiating studies with a mid-point prior to 1995 and those including and post January 1995. Exploratory analysis revealed that other regions did not have a significant time interaction. The possibility of using a continuous time variable was considered but decided against, due to unrealistically low 2008 estimates of diarrhea mortality as a result of linear extrapolation of the reduction observed during the 1990s.

We sought to include studies from all regions of the world, but failed to identify any studies from the WHO European region, a region that now includes several middle-income countries without complete vital registration data. For the purposes of the model, we substituted the Americas regional variable for these selected countries because this region had the next highest GNI and GNI proved to be an important predictor in the model.

We generated country level proportionate mortality estimates using a predictive logistic regression model for 84 countries without vital registration data (Table 1). The study level model was fit to data from all studies meeting inclusion and exclusion criteria. All studies contributed a relative weight to the final model results determined by the square root of the study sample size. We then estimated the regional proportion of diarrhea deaths (for countries included in this model) by taking the mean of the predicted country level proportions weighted by the total number of deaths among children under 5 years of age.

Regional level uncertainty bounds were estimated in a manner similar to that described previously [9] using a combination of leave-one-out cross-validation and Monte Carlo simulations. Each study was removed in turn and the model fit to the remaining studies. The out-of-sample prediction error distribution was estimated by the differences between the observed and predicted mortality rate log odds. Random draws from these regional prediction error distributions were used to perturb country estimates. Regional estimates were then calculated from the perturbed country level estimates and the process repeated 100,000 times. The regional 95% confidence interval is given by the 2.5^th^ and 97.5^th^ centiles. This uncertainty procedure only makes use of parameter point estimates and does not depend on the model parameter standard errors, which are likely to be underestimated due to correlation between studies from the same country or region. It should be noted, however, that the procedure does not account for all potential sources of variability, such as error in the estimates of total under 5 deaths. Analysis to identify overly influential observations using DFBetas and Cook's Distance metrics revealed two potential outlier studies [10], [11]. A decision was made not to exclude these studies as leaving them out had little influence on regional estimates.

We compared our country and regional specific diarrhea proportionate mortality estimates with those recently published for 2008 [5]. In brief, these estimates were developed using a multi-cause model for all countries except India and China [2]. The previously published Indian estimate was developed from a multi-cause model based on vital registration data from the Indian Sample Registration System (5). For China a series of single cause models were used based on verbal autopsy data available in the Chinese literature [12].

All statistical analyses were carried out using R version 2.13 [13].

The funding source had no involvement in the study design, analysis, or interpretation of results.

## Results

**Figure 1:**
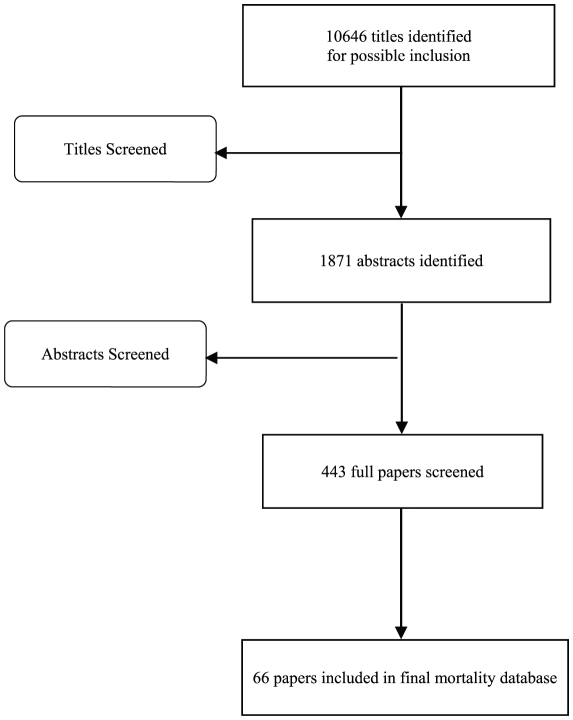
Systematic review and data abstraction flow chart.

**Figure 2:**
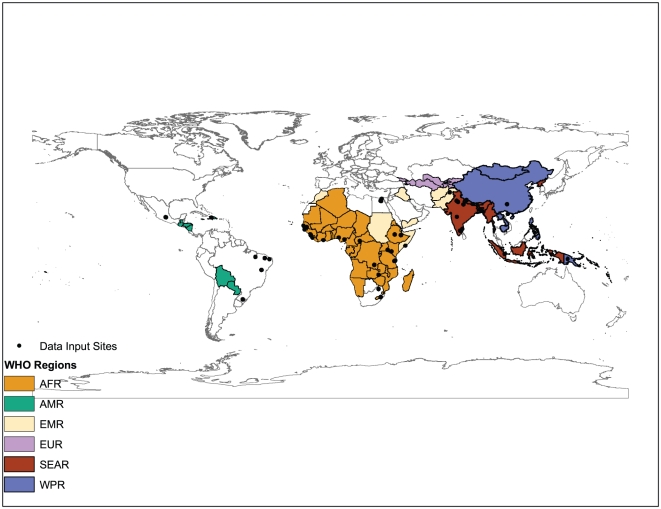
Geographic distribution of model input data and countries for which the model predicts proportionate mortality.

**Figure 3:**
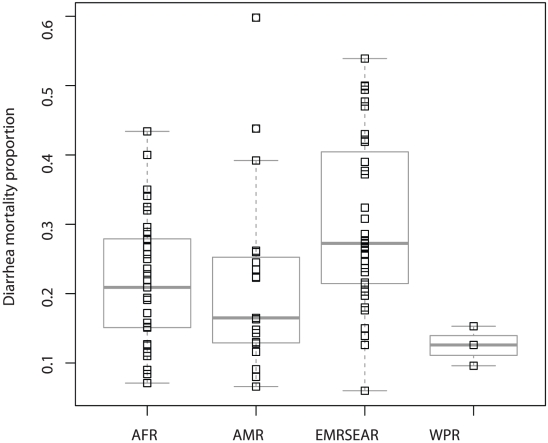
Distribution of study level diarrhea proportionate mortality by WHO region.

We reviewed more than 10,000 titles and included 66 studies representing 90 data points in our final review (Figure 1) (Web-Appendix contains full reference list). While we reviewed several unpublished data sources as part of the search, none provided the full scope of the methods and/or permitted inclusion in this review until publication. The data included represent all WHO regions of the world except the European Region (Figure 2). The distribution of study level proportionate mortality data are presented by region in Figure 3. We abstracted key study descriptors as well as 5q0, diarrhea proportionate mortality, and methods for obtaining cause of death.

The data were used to estimate proportion of deaths among children 1–59 mo of age in 84 low- and middle-income countries. Overall the countries included in this model represent 92–99% of the deaths among 1–59 mo old children in Africa, South-east Asia, Eastern Mediterranean, and the Western Pacific regions (Table 2). The modeled mortality includes a minority of countries and deaths in the Americas and Europe and hence is less generalizable in these regions. Consequently we do not make an estimate of the global proportion of deaths due to diarrhea.

**Table 2:** 2008 Diarrhea Proportionate mortality among children 1–59 mo of age among countries* without vital registration data compared to regional estimates from a multi-cause mixed model approach.

	Single-Cause			Multi-cause Mixed Methods Approach [5]
WHO region	Weighted Mean Diarrhea-Proportionate Mortality (%) [number of data points contributing to the estimate]	Uncertainty range	% of all deaths represented by the countries included in the model	Diarrhea-Proportionate Mortality (%)**
Africa^a^	25.2 [33]	20.2, 34.7	98.1	25.3
Americas^b^	10.0 [19]	6.6, 15.7	28.6	13.5
Eastern Mediterranean^c^	30.4 [36]	20.9, 47.1	91.6	30.9
Europe^d^	10.6	6.5, 17.2	39.2	10.6
South-East Asia^e^	31.3 [36]	18.0, 60.3	99.0	26.1
Western Pacific^f^	11.0 [3]	5.9, 15.3	94.1	8.3

*Countries Included in Single Cause Model: a) Algeria, Angola, Benin, Burkina Faso, Burundi, Cameroon, Cape Verde, Central African Republic, Chad, Comoros, Congo, Cote d'Ivoire, Democratic Republic of the Congo, Equatorial Guinea, Eritrea, Ethiopia, Gabon, Gambia, Ghana, Guinea, Guinea-Bissau, Kenya, Lesotho, Liberia, Madagascar, Malawi, Mali, Mauritania, Mozambique, Namibia, Niger, Nigeria, Rwanda, Sao Tome and Principe, Senegal, Sierra Leone, Swaziland, Tanzania, Togo, Uganda, Zambia, Zimbabwe; b) Bolivia, Dominican Republic, Guatemala, Haiti, Honduras, Jamaica, Nicaragua, Paraguay; c) Afghanistan, Djibouti, Iraq, Morocco, Pakistan, Somalia, Sudan, Yemen; d) Azerbaijan, Georgia, Kyrgyz Republic, Tajikistan, Turkmenistan, Uzbekistan; e) Bangladesh, Bhutan, DPR Korea, India, Indonesia, Maldives, Myanmar, Nepal, Timor-Leste; f) Cambodia, China, Lao, People's Democratic Republic of Micronesia, Mongolia, Nauru, Papua New Guinea, Philippines, Samoa, Solomon Islands, Vanuatu.

**100% of deaths included in multi-disease mixed approach Multi-cause mixed methods approach. Uncertainty ranges for total number of diarrhea deaths from MC model have been previously presented [5].

Regional differences are large with proportionate mortality ranging from 10.03% (uncertainty range: 6.6 to 15.7%) in the Americas to 31.3% (uncertainty range: 18.0 to 60.3%) in the Southeast Asian regions. Diarrhea proportionate mortality rates were greater than 25% in the African, South-east Asian, and Eastern Mediterranean regions, where nearly 9 out of 10 child deaths occur.

**Figure 4:**
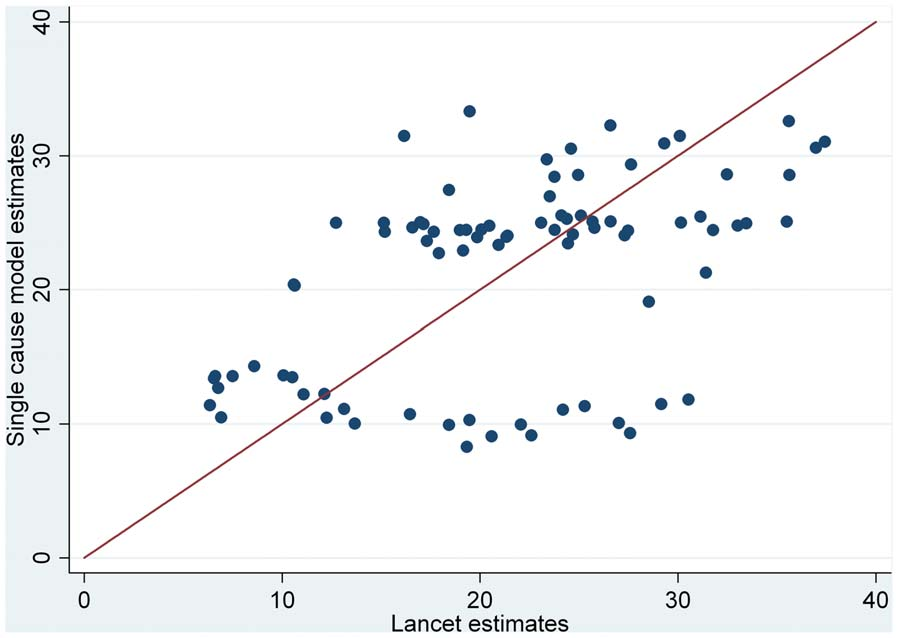
Comparison of country specific diarrhea-proportionate mortality estimates by country for the single cause-model vs. Lancet estimates [**5**].

We compared the country level results of the single-cause model with previously published results for 2008 and present both sets of estimates for proportionate mortality and deaths in Table S1 [5]. The country-level correlation coefficient is 0.5 (Figure 4).

## Discussion

In this paper we estimate that diarrhea was responsible for a varying proportion of deaths among children 1–59 mo of age in 2008 in major world regions. We did not calculate diarrhea deaths among neonates thus are unable to calculate the proportion of diarrhea mortality among all children under 5 years of age. Diarrhea deaths during the neonatal period have been estimated to be 1% of all deaths among children 0–59 mo of age [5]. We used a single cause modeling approach to project onto 84 countries without vital registration data. As might be expected, in regions where overall child (1–59 mo) mortality rates remain high diarrhea continues to account for a large proportion of child deaths, whereas in regions where overall mortality for children 1–59 mo of age is lower, case fatality rates have declined and thus diarrhea mortality rates have declined substantially. This review continues the CHERG standard of transparent methods and builds upon the first review estimating diarrhea proportionate mortality for 2000 [4].

This method ensures continued comparability across diseases for the single cause modeling approach and provides a comparative approach to the updated 2008 cause of death estimation process that included a verbal autopsy multi-cause model for a selected group of countries without vital registration data [5]. Our estimates for the South-east Asian and African regions, where the model includes countries accounting for 99 and 98% of the deaths respectively, are similar to the multi-cause model estimate for 2008 [2]. We estimated diarrhea was responsible for 31.3% of deaths among children 1–59 mo of age compared to the multi-cause estimate of 26.1% in South-east Asia. In Africa we estimated diarrhea accounted for 25.2% of deaths as compared to multi-cause estimate of 25.3% of deaths in this same age group. While differences in modeling approaches and input data result in wide variation for some country specific estimates, the similarities at the regional level provide confidence that a single cause modeling approach can be used to provide accurate estimates if the data are robust.

We chose the wide publication dates because a significant number of child mortality studies, especially those with quality diarrhea mortality data were published pre-1990 and while overall child mortality has declined, diarrhea incidence rates have not, thus we felt maintaining a wide net for potential studies would provide the best data possible. The model we present here represents more than 650,000 child deaths, yet despite this improvement in overall data availability when compared to previous estimates [4]; data remain limited from many regions. For example, for the former Soviet States where vital registration systems still do not cover at least 85% of child deaths, there are also no published child mortality studies and suitable data have not yet been identified in the unpublished literature for these countries [5]. Given that there were no studies identified from this region we used a regional dummy variable from another region, here the Americas, for modeling purposes. This geographic inequality with regard to the dispersion of data will continue to be a limitation in cause of death modeling. Until vital registration systems are in place in all low and middle income countries, a continued effort to generate cause of death data from countries and regions where information is currently lacking should continue to be a global health priority.

We included India and China in our regional estimates for 2008 diarrhea proportionate mortality. Given our literature review search strategies we identified a number of studies from both countries. However, in the recently published 2008 cause of death analysis these two countries were modeled separately and not included in regional models [5]. Because our goal was to produce regional estimates with corresponding uncertainty ranges, we chose to include them here as part of their respective regions and not to generate country specific models. We recognize the inherent limitations when including large countries in a global model as compared to more tailored country specific models where regional variations at the state or province level can be included. However, these estimates provide an opportunity for comparison and because these countries are important to their respective regions, their presence in our final model is important.

Because overall child mortality and the proportion of deaths occurring during the 1–59 mo age group relative to neonatal deaths continue to decline it is expected that the proportion of deaths attributable to diarrhea will also continue to decline. In the 2008 model presented here we included 33 new data points (90 in current review compared to 57 in 2004) and now include 4 studies with data collection periods in the 2000's [4]. The 2008 estimate presented here does not account for deaths in the neonatal period which we believe is a strength of the current analysis. In lieu of age adjustment, we used dummy variables to account for differences in reporting for studies that included vs. those that excluded neonatal deaths. We believe this strengthens the current regional estimates because the causes of death differ among neonates as compared to children 1–59 mo of age. As overall under 5 mortality declines and the ratio of neonatal to child deaths increases, thus it is increasingly important to address the causes of neonatal mortality separately. Our 2008 model tested several additional covariates as compared to the 2004 model. The use of dummy variables to adjust for studies with truncated age cohorts can be considered an improvement upon previous attempts at age adjustment with data only available from very few studies to generate age-specific mortality curves.

Relying on published data to estimate proportionate mortality has a number of inherent weaknesses. By nature of using retrospective verbal autopsy to assign cause of death, measurement error is possible. Though not perfect, verbal autopsy techniques have been published and standard algorithms exist to provide the best possible data in areas where physician confirmed cause of death are not available. A number of studies included in our analysis are from the 1980s and 1990s and are lacking site-specific data such as population specific 5q0 and are too old to successfully track down additional information from authors. When these data were not available we used the best national data corresponding to the time and place of data collection, but often the identified 5q0 was from another year and only available at the country level whereas more site-specific data was desired. In addition, we would have liked to have been able to abstract a number of diarrhea specific indicators from the studies themselves for use in the single cause model such as site specific diarrhea prevention and treatment variables, but these data are rarely reported in verbal autopsy studies and using national level proxy indicators to describe extremely variable behavior and treatment practices is far from ideal. In early models we tested a number of these variables and found that because most were not available from the data sources, they created noise in the final model and thus were deliberately excluded. If these data were available from the study sites and reported in conjunction with the diarrhea proportionate mortality rates this may enhance the ability of models to predict diarrhea mortality in countries where data gaps remain.

The estimates we report here represent an overall improvement in the available data with an increase in the total number and geographic distribution of data points and the inclusion of more recent mortality reports as compared to previous single cause estimates. As newer data are released we will be able to capture the most recent mortality trends with regard to overall rates as well as cause of death for children less than 5 years of age. With new prevention and treatment interventions such as insecticide-treated bednets, pneumococcal and rotavirus vaccines and zinc for the treatment of diarrhea being scaled-up in low- and middle-income countries, it will become increasingly important to capture the effect of these interventions on all cause and cause-specific mortality. There will inevitably continue to be a time lag from when interventions are successfully rolling out and saving lives to when data are available, but with continued emphasis on the critical importance of these data we are hopeful this distance from intervention to availability of mortality estimates will decline.

Despite recent reductions in all cause mortality, diarrhea remains the 2^nd^ leading cause of death among children under 5 years of age [5]. Our estimates are consistent with those previously published for 2008 based on a multi-cause verbal autopsy model and country specific data for India and China. These results should provide further justification for the continued effort to scale- up diarrhea prevention and treatment interventions targeted to children under 5 years. It is critical that countries with the highest burden of child mortality continue to target diarrhea deaths if the 4^th^ Millennium Development Goal of reducing child mortality by two-thirds is to be met by 2015.

## Supporting Information

Table S1 **Country level comparison of single-cause logistic regression and multi-cause mixed model approach.**
(DOCX)
