# Supplementary material for: Estimating Diarrhea Mortality among Young Children in Low and Middle Income Countries
Source: PLoS One. 2012 Jan 3;7(1):e29151. doi: 10.1371/journal.pone.0029151 (PMC3250411; doi:10.1371/journal.pone.0029151)
Supplement: Table S1 — Country level comparison of single-cause logistic regression and multi-cause mixed model approach. (DOCX) [file pone.0029151.s001.docx]

**Supporting Table 1. Country level comparison of single-cause logistic regression and multi-cause mixed model approach**

|  | **Single Cause** |  | **Multi-Cause Model** |  |
| --- | --- | --- | --- | --- |
| **Country** | **% of Deaths Attributable to Diarrhea** | **1-59 mo Diarrhea Deaths** | **% of Deaths Attributable to Diarrhea** | **1-59 mo Diarrhea Deaths** |
| **Africa** |  |  |  |  |
| Algeria | 19.2 | 708 | 28.5 | 3688 |
| Angola | 21.7 | 28003 | 31.4 | 40542 |
| Benin | 25.1 | 7057 | 17.7 | 4966 |
| Burkina Faso | 25.3 | 23118 | 23.8 | 21720 |
| Burundi | 26.4 | 8745 | 31.1 | 10313 |
| Cameroon | 24 | 16206 | 20.9 | 14139 |
| Cape Verde | 23.3 | 35 | 17.9 | 27 |
| Central African Republic | 25.9 | 4839 | 23.1 | 4312 |
| Chad | 25.2 | 19288 | 27.5 | 21030 |
| Comoros | 25.3 | 348 | 31.8 | 437 |
| Congo | 23.5 | 2640 | 19.1 | 2150 |
| Cote d'Ivoire | 24.7 | 12325 | 19.9 | 9908 |
| Democratic Republic of the Congo | 26.5 | 103572 | 25.1 | 98108 |
| Equatorial Guinea | 9.8 | 243 | 12.2 | 304 |
| Eritrea | 25.9 | 1866 | 30.2 | 2173 |
| Ethiopia | 26 | 51958 | 35.5 | 70944 |
| Gabon | 14 | 270 | 8.6 | 166 |
| Gambia | 25.3 | 1056 | 20.1 | 837 |
| Ghana | 25.1 | 8045 | 15.2 | 4868 |
| Guinea | 25.3 | 9434 | 19.3 | 7200 |
| Guinea-Bissau | 26.2 | 2425 | 24.4 | 2257 |
| Kenya | 24.8 | 34446 | 27.3 | 37934 |
| Lesotho | 24.4 | 575 | 17.3 | 408 |
| Liberia | 26.5 | 3685 | 24.1 | 3353 |
| Madagascar | 25.6 | 12063 | 33.0 | 15558 |
| Malawi | 25.9 | 10141 | 15.1 | 5927 |
| Mali | 25.5 | 18345 | 25.8 | 18548 |
| Mauritania | 24.1 | 1811 | 24.4 | 1836 |
| Mozambique | 25.9 | 18869 | 17.0 | 12373 |
| Namibia | 20.6 | 287 | 10.6 | 148 |
| Niger | 26 | 24457 | 25.7 | 24159 |
| Nigeria | 24.9 | 193836 | 24.7 | 192178 |
| Rwanda | 25.8 | 6976 | 33.5 | 9044 |
| Sao Tome and Principe | 24.8 | 81 | 21.4 | 70 |
| Senegal | 24.7 | 8126 | 21.3 | 7017 |
| Sierra Leone | 26 | 8505 | 26.6 | 8699 |
| Swaziland | 20.7 | 463 | 10.6 | 237 |
| Togo | 25.8 | 3345 | 17.1 | 2223 |
| Uganda | 25.6 | 37116 | 20.5 | 29677 |
| United Republic of Tanzania | 25.5 | 29508 | 16.6 | 19185 |
| Zambia | 25.3 | 14486 | 19.0 | 10869 |
| Zimbabwe | 25.9 | 6430 | 12.7 | 3158 |
| **Americas** |  |  |  |  |
| Bolivia | 8.7 | 667 | 27.0 | 2070 |
| Dominican Republic | 9.7 | 309 | 20.6 | 657 |
| Guatemala | 8.7 | 926 | 27.6 | 2937 |
| Haiti | 9.7 | 1206 | 30.5 | 3796 |
| Honduras | 8.9 | 270 | 19.5 | 590 |
| Jamaica | 11.6 | 139 | 19.3 | 231 |
| Nicaragua | 10 | 208 | 16.5 | 343 |
| Paraguay | 7.8 | 154 | 18.4 | 365 |
| **Eastern Mediterranean** |  |  |  |  |
| Afghanistan | 11.1 | 27485 | 35.6 | 88232 |
| Djibouti | 10.4 | 144 | 29.3 | 404 |
| Iraq | 9.6 | 1773 | 25.0 | 4610 |
| Morocco | 11.3 | 961 | 32.5 | 2762 |
| Pakistan | 9.6 | 17351 | 37.0 | 66821 |
| Somalia | 10.8 | 5611 | 30.1 | 15636 |
| Sudan | 28.9 | 24803 | 16.2 | 13870 |
| Yemen | 33 | 9753 | 37.4 | 11059 |
| **Europe** |  |  |  |  |
| Azerbaijan | 28.7 | 878 | 22.6 | 691 |
| Georgia | 30.2 | 187 | 13.7 | 85 |
| Kyrgyz Republic | 31.5 | 799 | 25.3 | 642 |
| Tajikistan | 31.1 | 2399 | 29.2 | 2249 |
| Turkmenistan | 29.8 | 898 | 22.1 | 665 |
| Uzbekistan | 28.9 | 2979 | 24.2 | 2493 |
| **South-east Asia** |  |  |  |  |
| Bangladesh | 27.6 | 19059 | 26.6 | 18360 |
| Bhutan | 28.9 | 195 | 23.8 | 161 |
| Democratic People's Republic of Korea | 34.2 | 3035 | 23.4 | 2074 |
| India | 33.4 | 275904 | 24.6 | 203206 |
| Indonesia | 31.2 | 28983 | 27.6 | 25664 |
| Maldives | 32.1 | 29 | 18.4 | 13 |
| Myanmar | 32.1 | 23896 | 19.5 | 14497 |
| Nepal | 27.1 | 3860 | 35.6 | 5072 |
| Timor-Leste | 31.7 | 641 | 23.5 | 476 |
| **Western Pacific** |  |  |  |  |
| Cambodia | 12.8 | 2699 | 10.1 | 2123 |
| China | 10.5 | 17176 | 6.4 | 10392 |
| Lao People's Democratic Republic | 12.6 | 853 | 10.5 | 712 |
| Micronesia (Federated States of) | 9.5 | 6 | 6.9 | 5 |
| Mongolia | 11.8 | 160 | 6.8 | 92 |
| Nauru | 12.7 | 0 | 6.6 | 0 |
| Papua New Guinea | 12.7 | 1100 | 7.5 | 650 |
| Philippines | 11.3 | 4517 | 12.1 | 4852 |
| Samoa | 10.2 | 6 | 13.1 | 8 |
| Solomon Islands | 12.5 | 41 | 6.6 | 21 |
| Vanuatu | 11.3 | 15 | 11.1 | 15 |
